# Precision prevention and the temporal disruption of evidence: the case of heart rate notifications from wearables

**DOI:** 10.1007/s11019-025-10308-0

**Published:** 2025-11-01

**Authors:** Sara Green, Christoffer Bjerre Haase, Olivia Spalletta

**Affiliations:** 1 Section for History and Philosophy of Science, Department of Science Education, University of Copenhagen, Niels Bohr Building (NBB), Jagtvej 128, 2200 Copenhagen N, Denmark; 2 Section for Health Services Research, Department of Public Health, University of Copenhagen, Oester Farimagsgade 5, 1014 Copenhagen, Denmark

**Keywords:** Precision prevention, Wearables, Wellness technologies, Medicalization, Evidence

## Abstract

Precision prevention refers to the use of data-intensive technologies to detect early indicators of disease and risk factors at the individual level. Precision prevention is not just a policy vision for a distant future but a development currently gaining momentum through wearables and self-tests marketed directly to consumers. We critically analyze one of the applications already on the market, namely detection of asymptomatic atrial fibrillation via smartwatches. We examine the promises made by manufacturers of smartwatches in relation to perspectives of general practitioners (GPs) in Denmark, who experience that new opportunities for disease prevention often come with new challenges. As one informant termed it, heart rate notifications are a form of “unauthorized screening” with uncertain benefits for individual patients and the healthcare system. The case of device-detected asymptomatic atrial fibrillation illustrates how precision prevention, via wellness technologies, can lead to a *temporal disruption of evidence.* We use this term to highlight the concern that evidence becomes the *result* of implementation, rather than the basis for it, thus turning consumers into experimental research subjects. The case of heart rate notifications also illustrates how the *proactive* approach to disease prevention, promoted by the wellness technology industry, drives a need for *reactive* research evaluating the benefits and harms of detection after the fact. We call for more attention to how big tech expansionism impacts the organization of health care and health research, as well as how the wellness technology industry shapes our understanding of disease and health.

## Introduction: the wellness industry and the future of medicine

Personalized or precision medicine (henceforth PM)^1^ has primarily gained attention as a strategy to offer tailor-made treatments to sick patients (e.g., Kerr et al. 2021; Tabery 2023). However, PM also entails a vision of *precision prevention*, understood as the expectation that data-intensive technologies can detect early, subtle signs of disease and disease risk, potentially improving disease prevention. The *All of Us* research program in the United States, for example, stresses that mapping of polygenic risk scores can improve disease prevention through more individualized or stratified approaches.^2^ Similarly, in European contexts, precision prevention has been framed as a strategy to reduce disease incidence and bring down rising healthcare costs. For example, the first action plan for PM in Denmark highlighted that “cheap and fast DNA sequencing will, in the coming decades, lead to completely new forms of individualized treatment and lifelong prevention” (Danske Regioner 2015a: 5, our translation). More recently, this vision has been expanded to also draw on the citizens’ own data from wearables (Danish Life Science Cluster, Deloitte & Enversion 2022). Precision prevention thus goes further than traditional health promotion strategies in engaging entire populations in data-intensive health monitoring and risk assessment.

Precision prevention is not merely a futuristic policy vision for public health but is already taking shape through consumer wearables and self-tests, as well as industry-sponsored health research. The world’s largest tech companies see health as their next big market, and companies like Apple, Huawei, and Fitbit, owned by Google’s parent company Alpha, are all investing heavily in user-generated health data. Through marketing of health benefits via wearable technologies, such companies are “transgressing spheres” that were typically seen as outside their domain, such as healthcare and health research (Sharon 2021a, b). At this intersection of digital capitalism and digital health, wearables are entering clinical practice as means for disease monitoring and prevention.

The development of wearable technologies for everyday use has also created opportunities for big tech companies to carry out health research through “pragmatic trials” such as the Apple Heart Study (Perino et al. 2021), the Fitbit Heart Study (Lubitz et al. 2021), and the Huawei Heart Study (Guo et al. 2019). A “pragmatic trial” refers to a study design operating in real-world settings, with minimal disruption to participants’ daily lives, and with the aim of producing results that are applicable to everyday practices (as opposed to clinical trials with highly controlled conditions). Pragmatic trials are promoted as more inclusive and with capacity to enroll participants at the scale of hundreds of thousands. In the context of company-driven biomedical research, such trials have been associated with concerns about privacy and data protection (Morain et al. 2022),^3^ but the implications and risks associated with big tech expansionism also point toward broader issues. These include how “technological solutionism”, driven by powerful tech companies, impacts the organization of health care and health research (Morosow 2013; Sharon and Gellert 2023). In this paper, we focus on how health benefits and risks are distributed and documented, as well as how the marketing of wellness technologies – more generally - shape our understanding of disease and health.

Wellness technologies refer to wearables and self-tests that are not classified as medical equipment for diagnostics but have monitoring, screening, detection, and prediction for health optimization in focus (Canali et al. 2022). This market is large because consumers can be both individuals with known illnesses and anyone wishing to improve their health and avoid future illness. The classification of these products as wellness technologies means that they do not face the same regulatory demands for documentation of health benefits as medical devices, screening programs, or diagnostic tests. Traditionally, implementation of a new test or screening program requires not only documentation of the *analytical validity* of a test (does the test accurately and reliably detect the risk factor or condition?) but also the *clinical utility* (does identifying the risk factor or condition lead to effective interventions that improve health outcomes?) (Green and Vogt 2016). But while public health strategies are supposed to be evidence-based, the wellness technology industry operates in a regulatory grey zone, where the benefits of health information and lifestyle changes are often taken for granted.

To exemplify, a pilot project called the *P100 study* involving 108 asymptomatic participants was promoted as a study in “scientific wellness” that demonstrated the benefits of intensive risk profiling and lifestyle counseling to reduce risk factors at the individual level (Price et al. 2017). The study included collection of billions of data points for all participants and counseling sessions with health coaches to optimize health through lifestyle changes. Yet, despite these positive results, it was criticized for drawing unwarranted conclusions of benefits from weak evidence, as the study lacked a control group and only measured relative reduction of risk factors over a short period (Vogt et al. 2018). Critics voiced concerns that the P100 study and similar pilot studies did not have - and are *not required* to have - a study design that allows for a proper evaluation of benefits and harms (Vogt et al. 2019). In response to critics, the PIs of the P100 study challenged the relevance of discussing possible harms in this context, as the study was primarily intended as a study in “scientific wellness” (Magis et al. 2018). The authors highlighted that interventions amount to identification of “actionable possibilities” for health optimization, e.g., risk factors or predisease categories pointing to benefits of lifestyle changes, rather than medical diagnoses and medical treatment. Moreover, the authors indicated that uncertainty about long-term benefits was not a limitation or cause for prudence but signified the need for longer studies with more participants - as exemplified through their subsequent 100 K project, which was initiated via their DTC company Arivale (see also Hood and Price 2023).^4^

Our paper is motivated by cases such as the P100 study, which highlight the need for further reflection on the increasing role of the *wellness technology industry* in developing, implementing, and promoting precision prevention strategies. While strategies to improve lifestyle and optimize health may seem harmless, the results of the P100 study expose the risks of overmedicalization and overdiagnosis of healthy people, as *all* participants were diagnosed with risk factors or predisease, such as prediabetes (Vogt et al. 2018).^5^ Overdiagnosis arises from the uncertain causal relationship between detection of abnormalities (e.g., risk factors) and actual disease development. Because many abnormalities will not lead to symptomatic disease, if left untreated, screening for risk factors or early signs of disease always comes with a potential tradeoff between the capacity of improved prevention in some cases and harmful overdetection and overtreatment for others (Plutynski 2012; Brodersen et al. 2018). Even if the focus in precision prevention is often merely to promote a healthier lifestyle, potential harms include unnecessary worries, waste of healthcare resources for follow-up testing and counselling, as well as costs and possible side-effects from (unnecessary) preventive medical treatments, such as statins and blood thinners (see also Dumit 2012). Moreover, the intensified focus on identifying risk factors in all individuals has been associated with *medicalization*, understood as a process by which non-medical aspects of human life are increasingly considered part of the medical domain. While overdiagnosis and medicalization are different concepts, they are both associated with contested expansions of disease concepts, such as prediseases (Hofmann 2016, 2017). As precision prevention entails continuous measurement and repeated testing for risk factors, some scholars have interpreted these developments as a “technoscientific holism” entailing “medicalization of health and life itself” (Vogt et al. 2016).

Despite well-documented problems of overmedicalization and overdiagnosis in the history of medicine, it is curious to note that criticism and acknowledged uncertainties often do not motivate caution for implementation. Instead, the burden of evidence is often shifted onto the quantity of data currently available. In this context, uncertainties are actively used to call for upscaled and faster implementation to collect more data (see also Timmermans et al. 2017). This can be interpreted as a methodological and ethical reversal of how health research is typically justified. Validation here becomes the *result* of implementation, rather than forming the evidence basis for it, creating what Green et al. (2022) call a *temporal disruption of evidence*. We here draw on and further elaborate on this concept in exploring how the uptake of wellness technologies on the consumer market, more generally, may impact the organization of health care and health research. For this purpose, we combine methods from philosophy of science and medical anthropology, as clarified in the following section.

## Methods and context of the study

Individually and collectively, we have explored the implications of datafication or data-intensive resourcing (Ruckenstein and Schüll 2017; Hoeyer 2019) in Danish healthcare, focusing specifically on implications for general practice. From 2020 to 2025, the authors interviewed 43 Danish GPs in two parallel research projects, one focused on how GPs view PM and implications for disease prevention, and another on how GPs use data in their daily practice. Informants for both projects were recruited through professional organizations and via snowball sampling, and interviews lasted about 60 min on average. The interviews have been audio recorded, transcribed ad verbatim, and all translations from Danish to English were made by the authors. The research projects were approved by the ethics committee at the University of Copenhagen. The collection and use of empirical examples comply with the requirements of the EU’s General Data Protection Regulation (GDPR), and all informants have been anonymized in quotations used in the following section.

While the data were initially collected for two different research projects, common research interests and findings emerged from research seminars. We therefore explored opportunities for a joint analysis relating to the prospects and challenges of using citizens’ own data for improved disease prevention. In analyzing our material towards this aim, we have interpreted our respective material through an iterative process of coding for common themes, such as GPs experiences with self-reported data, and discussing our analytical ideas (Timmermans and Tavory 2012). As one of the often-mentioned applications was the case of heart rate notifications from smartwatches, we have decided to focus on that example here. Importantly, our aim in this paper is not to evaluate the clinical utility of wearable technologies in general. The functionality and accuracy of wellness technologies are rapidly improving, and wearables can have wide-ranging potential for both disease management and disease prevention, depending on their specific features and contexts of use. Our focus here is instead to explore the implications of the way precision prevention strategies, via wearables, currently materialize and enter public health via consumer markets. Our analysis is inspired by methods in medical anthropology and philosophy of science. We first unpack the complexity of this relationship by comparing manufacturers’ promises with interviews from Danish GPs, highlighting potential benefits but also clinical uncertainties associated with new detection possibilities. Moreover, we examine existing clinical evidence assessing the benefits and harms of screening for atrial fibrillation in asymptomatic individuals.

Denmark is an interesting location to explore the implications of wellness technologies not only for consumers but also for the public healthcare system. All residents have access to healthcare free of charge, and all are assigned to the care of a GP. In this context, concerns about distributive justice are not directly related to disparities in access to healthcare services, as is the case for insurance-based systems such as the healthcare system in the U.S. (cf. Fleck 2022a, b; Tabery 2023). Yet, wellness technologies may, in the Danish context, provide a threat to solidarity in other ways, e.g., by draining the collective healthcare resources (Green et al. 2023). GPs are relevant informants for such discussions, as they function as “gatekeepers” for access to many healthcare services, a function intended to protect healthcare from costly overuse and to prevent unnecessary and potentially harmful overtreatment of patients.^6^ As doctors to the nation, Danish GPs thus stand at the front line of preventive medicine in evaluating whether a health technology is beneficial. This is not merely a question of whether it benefits the individual patient. It also involves considerations about how to best prioritize healthcare resources and ensure that public health strategies are evidence-based and cost-effective.

The paper is structured as follows. We first describe the promises made by the manufacturers and the studies initiated to document the accuracy of heart rate monitoring via this technology (Sect. “Towards scientific wellness? The case of heart rate notifications”). We then turn to how practicing GPs view the prospect of early detection of atrial fibrillation via wearables and how they experience existing uncertainty about benefits (Sect. "Wearable technologies in general practice"). We further unpack this uncertainty by examining the results from both “pragmatic” and randomized controlled trials on early detection of asymptomatic atrial fibrillation (Sect. "The sooner, the better? The problem with overdiagnosis"). Section "The temporal disruption of evidence and the need for reactive research" elaborates on the concern of a temporal disruption of evidence in the context of pragmatic trials financed by big tech, where their *proactive* approach to disease prevention through intensified data collection creates a need for *reactive* research and follow-up care. Section "Concluding remarks" concludes with reflections on how the wellness technology industry may reshape how health is measured and defined.

## Towards scientific wellness? The case of heart rate notifications

A recent book by Hood and Price, the PIs of the P100 study and the Arivale company, claims that we are reaching the age of “scientific wellness”, where “the future of medicine is personalized, predictive, data-rich and *in your hands*” (Hood and Price 2023). The book explains how a proactive approach to health optimization, drawing on genomics, wearables, and AI, can revolutionize preventive medicine and empower users to reduce the development of disease and extend their lifespan. Similar visions have been promoted by the cardiologist Topol in the books entitled *The creative destruction of medicine* (2012), *The patient will see you now: The future of medicine is in your hands*, (2014), and *Deep medicine: How artificial intelligence can make healthcare human again* (2019). What these have in common is the emphasis on patient empowerment through access to more health information at the individual level, enabling greater control of health outcomes (Vegter 2018; Vegter et al. 2020; Kreitmair 2024). The expectations of impact and empowerment rest on the assumption that intervening earlier, before symptoms are experienced, can reduce the onset of health problems, thus improving both the efficacy and cost-effectiveness of disease prevention. Precision prevention is further premised on the idea that disease risk differs greatly among individuals, and that health optimization requires technologies measuring finer-grained signs of early disease at the individual level (Vegter et al. 2021).

**Fig. 1:**
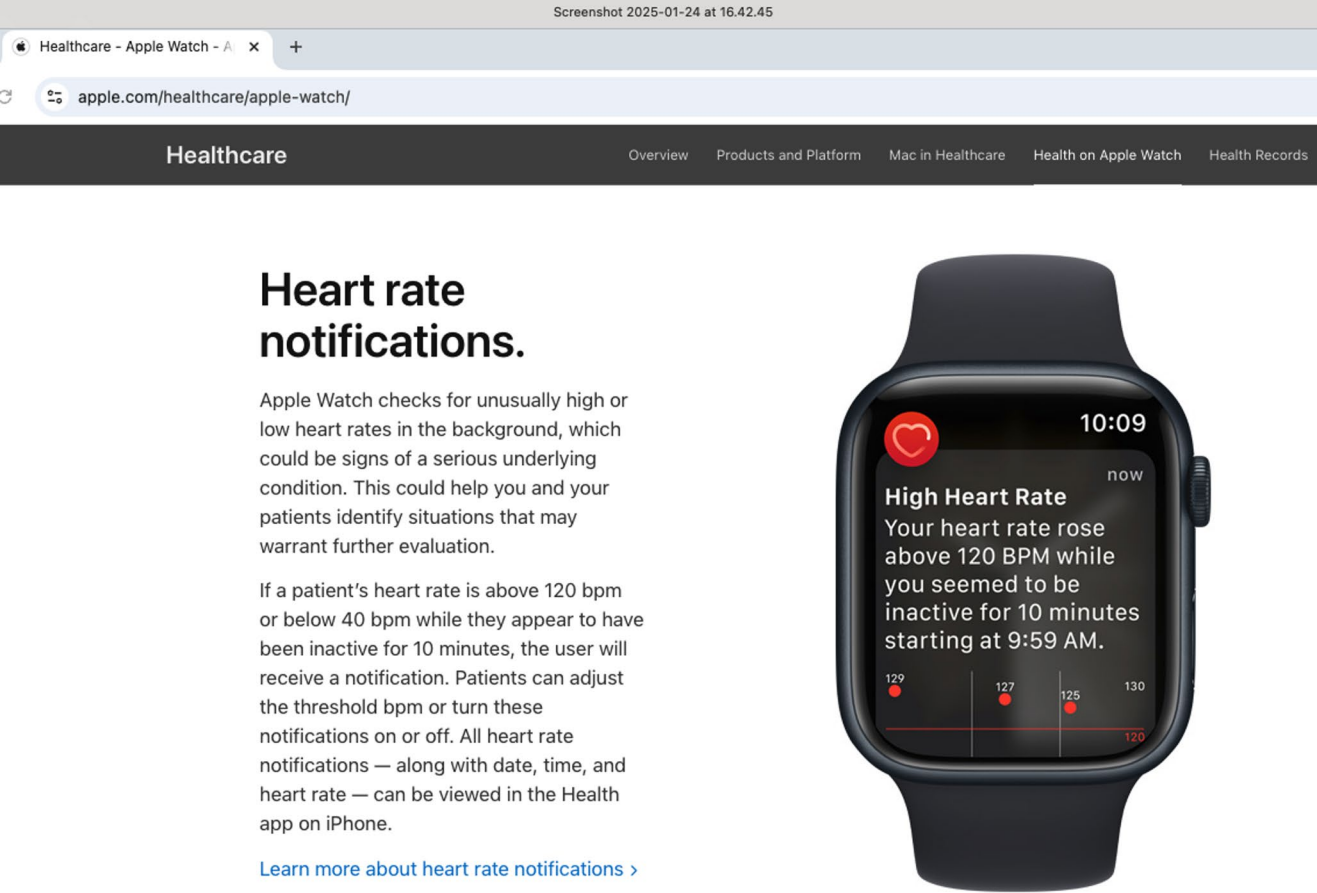
An example of information provided on the benefits of receiving heart rate notifications. Screenshot of Apple’s support webpage on “Heart health notifications on your Apple Watch”: https://support.apple.com/en-us/120276, accessed January 24, 2025

Companies marketing wellness technologies to consumers use similar rhetoric, and technologies developed by big-tech companies are increasingly entering both users’ homes and healthcare clinics (Sharon 2021a, b; Stevens 2024). By monitoring various bodily functions via smartwatches, users can become more aware of how their activity, stress, diet, etc., affect the body. For example, Apple’s website emphasizes that its smartwatch “empowers your patients to take control of their own health”, and a white paper from the same company describes Apple Watches as an “intelligent guardian of the user’s health” (Apple Health Report 2023, p. 9). The metaphor of an intelligent guardian of health captures how the watch continuously monitors the physiological state of the user and can identify the “invisibly sick.” These are individuals who feel healthy but have unknown health issues that can only be detected through continuous monitoring. Detection of asymptomatic heart problems is a prominent example. According to Apple’s webpage, irregular heart rhythm “may be suggestive of atrial fibrillation”, or AFib.^7^ The user is informed that “[i]f left untreated, AFib can lead to heart failure or blood clots that may lead to stroke. AFib can be managed with a doctor’s care and medication, and early diagnosis and treatment can prevent such complications.” Apple also caters to practicing health professionals by highlighting that “Apple Watch checks for unusually high or low heart rates in the background, which could be signs of a serious underlying condition. This could help you and your patients identify situations that may warrant further evaluation” (see Fig. 1).

Among the highlighted benefits are continuous data collection in the individuals’ everyday life and home, rather than “snapshot” measurements of heart rate and blood pressure at the doctor’s office. In contrast to tests performed in the clinic, devices like Apple Watches are mobile and always on, enabling more efficient identification of potential problems. Similarly, a report published by Apple stresses that “researchers and clinicians are able to accomplish even more with the addition of Apple Watch to their research and care programs” (Apple Health Report 2023, p. 27), and that their technologies allow “clinicians to be more mobile, leading to time savings and more time spent taking care of patients” (p. 38). Such reports are based on experiences where medical doctors have adopted Apple’s research initiatives in clinical practice, e.g., to finance or speed up digitalization of healthcare practice. Stevens (2024), drawing on Sharon (2021a, b), refers to this development as “sphere transgressions in medical research.” The term captures the growing influence of big-tech companies in various domains, including dependencies of public healthcare on specific commercial products and participation in the company’s research through user deals.

Partnerships between tech companies and healthcare systems are also increasingly embraced in policy reports as a way to make better use of health data. The benefits of detecting asymptomatic cases of atrial fibrillation are, for example, highlighted in a report commissioned by the Ministry of Health and the Ministry of Industry, Business, and Financial Affairs (Danish Life Science Cluster, Deloitte & Enversion 2022). The report presents a series of fictional ‘cases’ that offer a glimpse into what the future might look like. In one of these, a 60-year-old woman (Susanne) is notified by her smartwatch that her pulse and heart rhythm were outside the normal range during the night. Susanne initially ignores the notification, as she feels fine, but later she is called by a medical secretary who urges her to go to the nearest hospital as soon as possible. In this fictional example, Susanne is diagnosed with anemia and atherosclerosis, as well as asymptomatic atrial fibrillation, for which she now receives medical treatment. The report states that these conditions “would not have been discovered without her own data, and the early intervention has potentially prevented a stroke” (Ibid, p. 88).

The fictional example of Susanne is a good illustration of how counterfactual reasoning about what could have happened without data can motivate or speed up PM initiatives and investments. The report also reflects broader political expectations that data and digitization can and should solve many of the problems facing a strained social and healthcare system. In this context, intensified data sourcing and implementation of more technological solutions are often considered as a necessary development for the healthcare system. For example, the Danish Health Data Authority’s Strategy for Digital Health 2018–2024 asserts that there is “in reality no alternative to increased digital collaboration” between citizens and healthcare professionals (Sundhedsdatastyrelsen 2018, p. 8). Yet, as we clarify in the following, it is difficult to know whether the detection and treatment of asymptomatic atrial fibrillation (as in the fictive case of Susanne) is overall beneficial or harmful. Below, we unpack the challenges from the perspective of practicing Danish GPs (Sect. "Wearable technologies in general practice"), as well as an examination of results from trials concerning device-detected atrial fibrillation (Sect. "The sooner, the better? the problem with overdiagnosis"). We contend that insights from these sources add to discussions on the implications of technological solutionism, including questions about who is likely to be “empowered” through the use of said technologies (Sect. "The temporal disruption of evidence and the need for reactive research").

## Wearable technologies in general practice

General practice in Denmark is typically the first point of contact for most health-related concerns, and GPs serve as gatekeepers to many forms of diagnostic testing and secondary healthcare services. While Danish GPs have the responsibility to coordinate care for chronically ill patients, our informants described much of their work as mundane in the sense of checking and treating non-serious symptoms and conditions, performing routine care, listening to worried patients, and helping patients manage shifting concerns across the life course. When asked about the potential of precision prevention, including opportunities for self-monitoring via wearables, GPs would often distinguish between uses targeting patients with chronic symptomatic disease and promises of early detection in asymptomatic patients.

In the case of chronic disease management, some GPs explained that technologies for continuous self-measurement of heart rate, blood pressure, and blood glucose can help them monitor a patient’s condition with higher precision, as they get insights into how the patient’s health parameters vary over the course of hours, days, or weeks. This could help them provide care more efficiently, e.g., by adjusting the dosage of prescription medicine or reducing unnecessary control consultations. Yet, for patients *without* current health problems, GPs in large part resisted the notion that the current slate of precision prevention offerings –such as genetic self-tests or self-monitoring to evaluate risk of future disease – offered actionable information. On the contrary, GPs told us that this kind of information might unnecessarily worry or confuse patients.

While self-monitoring data for GPs can give useful additional information about the context of the patients’ symptoms (e.g., activity levels compared to experienced symptoms), they often found it challenging to convey to patients when they should react and contact the GP after receiving such information. Several GPs reported that patients did not always understand that it is normal for their blood pressure or heart rate to fluctuate during the day, and that minor irregularities over short periods should not be a cause of concern. Wellness technologies thus introduce new didactical challenges in primary care, as interpretation of health data from wearables may require new forms of digital health literacy.^8^ Moreover, GPs balked at the notion that consumer-grade tests and devices would enact prevention by inspiring patients to make lifestyle changes based on risk profiling. This, GPs told us, ran counter to what years of practice had shown them, i.e., that staying motivated to live healthier is difficult for most people, even if patients know that their individual risk is high.

When GPs were asked whether and how wellness technologies affect their clinical practice, several mentioned the example of wearable devices detecting irregular heart rhythm, which raised concerns for patients about asymptomatic atrial fibrillation. As mentioned, smartwatches are not diagnostic devices, and the users had to consult their GP for follow-up testing and guidance. As one GP clarified:

I have had several patients coming with an alarm for atrial fibrillation on their watch. And unfortunately, I couldn’t use that diagnosis, because I didn’t find a way to document it in my system. So, I had to do the old school way: ECG in the clinic. We didn’t find anything. I sent them to the cardiologist, and they collected data from a Holter monitor over several days. Due to lack of documentation or lack of integration, I wouldn’t start a treatment [based] on something I couldn’t document.

The GP clarifies how existing guidelines bars them from diagnosing the patients based on smartwatches alone, which may lower concerns about overdetection due to lack of documented accuracy of wearables. Yet, the quote illustrates how the increasing use of wearables can lead to increasing follow-up testing in both primary and secondary care. While intensified testing may catch more medically relevant cases, the GP also explained that it is difficult to know whether the follow-up testing is beneficial, as not all cases of asymptomatic atrial fibrillation develop into symptomatic heart-related disease. The potential of catching more medically relevant cases, therefore, comes with the risk of overdiagnosing harmless abnormalities. Another GP put it this way:

If you have irregular heart rhythm, you can actually see a rise in the heart rate when it’s not relevant. That means you on this basis can suspect what we call atrial fibrillation. And from that you can move on to preventive treatment. [But] if the patient would never have developed a blood clot from that, this would have been wasted, right? And then there are all these issues about what it does to the patient, does the treatment we give benefit the patient? We cannot know that in advance.

The GPs’ guidelines state that if atrial fibrillation is documented, the patient should be offered blood thinners to prevent stroke. These guidelines are based on studies documenting benefits of blood thinners for patients experiencing symptoms, such as shortness of breath, fluttering heartbeat, or high pulse after activity. Indeed, some of the GPs we interviewed also emphasized that data from smartwatches can provide a clearer picture of what happens when patients experience such symptoms, as they allow for continuous monitoring outside clinical consultations. But they also explained that when an increasing number of asymptomatic individuals monitor their heart with increasing precision, new uncertainties arise about how many patients with irregular heart rhythm should be prescribed blood thinners, as potential benefits also come with associated risks such as bleeding risks (from easy bruising to hemorrhagic stroke). The uncertainty about benefits for asymptomatic patients presents a dilemma for practicing GPs, as clarified in the following quote:

I think atrial fibrillation is more sort of a spectrum of diseases and there are several patients, who have atrial fibrillation, that we do not have to treat. We need to find the right patients, and that’s the tricky part. […] We know that all the big tech giants can do, I call it, *unauthorized screening*. And it’s very interesting that patients can come into my clinic with the diagnosis, and I did not order any tests. I did not tell them to do anything, but they brought the diagnosis. And I think we will learn that many diseases have many different phases, and some go away by themselves.

What the GP here calls “unauthorized screening” refers to the marketing and use of devices not classified as diagnostic tools, but which nevertheless collect health data and notify patients about disease risk before the GP has had the opportunity to guide the patient or run any tests. As wearable devices advance monitoring of more people with increasing precision, more cases of irregular heart rhythm may now be detected in populations that typically would not be considered as being at risk of heart problems. Even when GPs experienced that a warning of possible atrial fibrillation was confirmed through ECG or tests at the hospital, they were quick to point out that they did not know whether this will be beneficial, considering the uncertainties and risks of side-effects from blood thinners.

As seen in the previous section, in marketing material and policy papers, wearables are envisioned as life-saving technologies that reduce both suffering and costs by alerting users of potentially serious health conditions. In serving this role, wellness technologies are marketed as devices that “empower” users to “take control” of their health (Kreitmair 2024). But while GPs acknowledge the possibility of catching a few potentially serious cases and making some patients more informed about their health, they stress that this often comes at the price of introducing new - and often unnecessary - worries for many:

Well, it’s easy to think that [self-monitoring] would be a way to address the patient’s worries. More often, it looks to me as if self-monitoring creates new kinds of worries for the patient. The ones that come and say that ‘My Apple Watch tells me that I have an irregular heartbeat.’ Or the ones that tell me that ‘My watch tells me that I’m not sleeping well.’ They’re telling me about new problems that they didn’t know of before. So, they’re worried about something that they wouldn’t be worried about if they didn’t have that kind of data. Sometimes, but not very often, it leads to finding some medical problem that we didn’t know of. But most of the time, it’s just a new kind of worry that we end up finding has no medical consequences. And that may even divert attention from something else that was perhaps more important to discuss.

Another GP similarly reflected on how data from wearables add a further layer to the complexity of health information in modern society, as the increasing accessibility of knowledge and measuring devices do not always result in more precise (self)diagnostics but can be challenging to navigate for patients and professionals alike. The GPs currently lack guidelines for clinical use of wearables, and several explained that they did not feel prepared for - or had time for - the request to help patients interpret such data. Such uncertainties and challenges highlighted by the GPs stand in a striking contrast to the marketing of wellness technologies online and promotion of self-monitoring as a solution to the healthcare crisis in policy reports. It is therefore relevant to ask what evidence is needed to confirm either optimistic expectations or concerns. This is the question we turn to in the following sections.

## The sooner, the better? The problem with overdiagnosis

It seems intuitive that it is better to prevent than to treat, and “the sooner, the better”, as a Danish health policy report on prevention of common disease is titled (Regeringen 2014). However, preventive medicine has historically faced difficult challenges in documenting health benefits, even when risk factors are successfully reduced. Currently, the European Society of Cardiology and the US Preventive Services Task Force do not recommend general screening for atrial fibrillation (Hindricks et al. 2020; US Preventive Task Force 2022). The increasing use of wearables for heart rate monitoring has therefore raised debate in the field about whether device-detected subclinical atrial fibrillation should be treated like symptomatic and clinical atrial fibrillation (Kalarus et al. 2023; Sanders et al. 2024; Salmon et al. 2024). A large Danish study called LOOP illustrates why.

In the LOOP study, a loop recorder (ILR) was implanted to detect atrial fibrillation, similar to the function of advanced smartwatches, and the detection rate and relative benefits of detection were compared to the normal procedure for diagnosing atrial fibrillation (Svendsen et al. 2021). In this study, 6004 participants aged 70–90 with existing risk factors for stroke were recruited for a randomized controlled trial with a 5-year follow-up period. Among patients who were randomly assigned to the ILR group and monitored continuously, 32% were diagnosed with asymptomatic atrial fibrillation, compared to about 12% in the control group. In both cases, standard treatment with blood thinners was initiated upon detection. However, even though continuous monitoring almost tripled the detection of atrial fibrillation, the study showed no significant reduction of stroke or systemic arterial embolism in the intervention group. Similarly, there was no significant difference in cardiovascular and overall mortality between the groups. As the study showed increased prescription of blood thinners, resulting in a modest increase in bleeding rates, the authors concluded that this may imply that “not all atrial fibrillation is worth screening for, and not all screen-detected atrial fibrillation merits anticoagulation” (Svendsen et al. 2021: 1507). To this one may add possible harms associated with unnecessary worries about the risk of serious heart conditions (not measured in the study).

The results of the LOOP study were a surprise to the researchers, who anticipated a stronger similarity between detection and treatment of atrial fibrillation detected during ordinary health checks and subclinical atrial fibrillation detected by devices. The finding that such patients did not benefit from blood thinners demonstrated that this was not the case. Thus, looking “harder and longer” for abnormalities via such devices will likely not only catch patients that would go on to experience symptoms, but also a broader spectrum of abnormalities with unknown implications for the individual’s current or future health (Kalarus et al. 2023). The findings of the LOOP study may expose the risks of what in the medical literature is called *spectrum bias*, i.e., the bias of uncritically extending diagnostic tests to patient populations different from those where they have documented utility (Ransohoff and Feinstein 1978). A study measuring the degree of overdiagnosis of atrial fibrillation of asymptomatic individuals through heart rate monitoring is currently under peer-review (Haase et al., under review). Aside from raising concerns about the risk of overdiagnosis, the author LOOP study also ​​underscored the need for high-quality clinical trials with extended follow-up periods in low-risk populations.

Meanwhile, the highly limited regulation of consumer devices has opened the door for companies to conduct health research following quite different evidence standards. For instance, in recent years, the *Apple Heart Study* enrolled 419,297 participants as research subjects in order to validate their devices for detecting atrial fibrillation (Perez et al. 2019; Perino et al. 2021). Users who received a notification from the Apple Watch about irregular heart rhythm were offered the possibility to undergo an ECG with a Holter monitor over several days, akin to the procedure described by a GP in the previous section. The purpose of the follow-up ECG was both to validate the device against standard clinical practice, which could then be marketed as an accurate tool for measuring heart rate and irregularities, and to satisfy concerns that users receiving notification were pointed towards more thorough follow-up testing (and encouragement to contact health professionals if the ECG confirmed atrial fibrillation).

Following the Apple Heart Study, concerns have been raised about the risk of false positive results, including a study retrospectively evaluating the rate of clinically actionable diagnosis in patients seeking medical attention after receiving heart rate notifications (Wyatt et al. 2020). While this is an important issue, the concerns motivated by the GPs’ experiences and the LOOP study are broader. In this context, improved accuracy (in terms of increased sensitivity and specificity) can paradoxically increase overdiagnosis as a result of overdetection (Vogt et al. 2019). This is particularly the case if a technology is used to detect fine-grained abnormalities in populations that would normally not undergo screening for heart-related problems. Yet, problems with overdetection and overdiagnosis are not the focus of the Apple Heart Study or similar pragmatic trials sponsored by big tech companies.

The Apple Heart Study was designed as a prospective, single-arm pragmatic trial (Perez et al. 2019; Turakhia et al. 2019; Perino et al. 2021). While this study design excels in requiring limited interruption in participants’ daily lives and reaching very large samples (several hundred thousand participants), it is not designed to evaluate whether study participants with identified atrial fibrillation and subsequent treatments benefit from this intervention. Instead, such trials are set up to document that the devices are *accurate* compared to medical devices (e.g., ECG) – a positive result that is now highlighted on their websites. But they are not designed to investigate the *clinical utility* of detecting asymptomatic atrial fibrillation in a general population, i.e., outside the guidelines of the medical devices. This would require a retrospective randomized trial evaluating the relationship between benefits and harms of device-detected abnormalities. Thus, the uncertainties about clinical benefits remain despite the scale of such studies (Salmon et al. 2024). Meanwhile, the responsibility and cost of diagnosing and treating users flagged by these studies fall not to manufacturers, but to patients, doctors, and their local healthcare infrastructures. Hence, the choice of Apple, Fitbit, and Huawei to focus on accuracy, rather than utility, creates a knowledge gap about the health benefits of the expanded market for monitoring and early detection.

The LOOP study was among the first to suggest that the benefits of continuous monitoring are likely to be a costly mirage, even among patients at risk for cardiovascular events. If the results from the study are indicative of overdiagnosis even in this group, there is reason to expect that the problem is higher for general screening.^9^ The GPs’ concerns and the results of the LOOP study underscore that the implied alliance between the wellness industry and primary care should not be taken for granted. Rather, there is a risk of GPs and cardiologists unwillingly end up facilitating increased overdiagnosis, because existing guidelines require prescription of preventive medicine if atrial fibrillation is confirmed through follow-up testing. In such cases, the burden of overdetection is placed on public healthcare systems, which also have to address the knowledge gap concerning the relative benefits and harms of device-detected subclinical atrial fibrillation. We elaborate on this concern in the following section, returning to the implication of the temporal disruption of evidence.

## The temporal disruption of evidence and the need for reactive research

Sections "Wearable technologies in general practice" and "The sooner, the better? the problem with overdiagnosis" highlighted how the benefits of detecting subclinical atrial fibrillation via wearable devices remain unclear. Follow-up research on the LOOP study resulted in the development of a new medical term, *subclinical atrial fibrillation*, i.e., a risk category identified and constructed via technological devices for continuous monitoring (Joglar et al. 2023; Kalarus et al. 2023). The current guidelines state that, as the risk of stroke for patients with device-detected subclinical atrial fibrillation is lower, the threshold for medical intervention is higher, and blood thinners may be unnecessary for most (see also Salmon et al. 2024). Nevertheless, in the current regulatory landscape, the wellness industry remains free to capitalize on the popular notion that early detection through continuous monitoring has the potential to save lives. Manufacturers of wellness technologies still market general screening for atrial fibrillation as beneficial, while avoiding responsibility for harms. This is because, unlike manufacturers of regulated medical devices or pharmaceutical drugs, consumer wellness technologies are not required to substantiate the scope of benefits (or harms) of detecting signs or symptoms of disease. Given that everyday screening can result in harm to both patients and healthcare systems, the basis for current legal distinctions in the regulation of health technologies and drugs deserves critical scrutiny.

At present, the relationship between benefits and accountability deliberately gets blurred in what is communicated to consumers. Companies selling wearables typically have disclaimers that the devices are for “educational purposes only” and not intended as diagnostic devices.^10^ Yet, while they are not approved to diagnose atrial fibrillation, they still market such devices as a “guardian of health” (Apple) that can “screen for potential health risks” (Huawei), thus sending the signal to users that they are safer when using the smartwatch. Moreover, despite the lack of approval as medical devices, the devices still provide users with heart rate notifications and an electrocardiogram (ECG) directly on their smartphone. The companies bear no responsibility for what is measured, as they advise users to see a doctor if they are worried about their data. We suggest that the premature implementation of “everyday” or “unauthorized screening” amounts to a *temporal disruption of evidence.* While, traditionally, interventions are developed based on clinical evidence demonstrating benefit, a temporal disruption of evidence reverses the order. Here, evidence is the result of large-scale implementation. Although users of these devices technically consent when they accept the terms, this turns consumers into experimental research subjects in new forms of health research - with unknown implications for citizens and healthcare systems.

In the current regulatory atmosphere, device manufacturers are thus not obligated to substantiate the promised benefits. Rather, wearable devices enter a market primed by public (and often political) expectations that early detection is always beneficial–an expectation manufacturers explicitly endorse and perpetuate. People who find themselves in the new risk category are perfectly placed as consumers of medical surveillance products. This not only changes how medical research is done, but also who pays the price. While there is little evidence that surveillance benefits healthy populations, the wellness industry benefits from transforming healthy bodies into voluntary research subjects and a market of potential lives “saved”, while displacing the burden and cost of care and potential harms from overtreatment. Meanwhile, although devices are sold to consumers not as approved medical devices but information that is “for educational purposes only”, the companies promote consumer medicine via self-monitoring as a new way of conducting medical research.

While these companies launch and sponsor their own clinical studies, it is currently unclear what the public returns of research participation are in contexts where “information commons” are developed and owned by for-profit companies (Prainsack 2019). We have seen how big tech companies recently have conducted large-scale trials involving hundreds of thousands participants. These trials are not designed to evaluate health benefits of early detection via self-monitoring devices but are investments for documenting accuracy and expanding market shares. The Fitbit Heart Study (Lubitz et al. 2021), the Huawei Heart Study (Guo et al. 2019), and the Apple Heart Study (Perez et al. 2019; Turakhia et al. 2019; Perino et al. 2021) were impressively large studies providing so-called “real-world evidence” through a pragmatic trial conditioned on enrollment via wide inclusion criteria. While the outcomes of such larger studies can provide useful insights into the spectrum of irregular heart rhythm conditions, the trials are primarily geared towards assessing and documenting the *accuracy* of the devices, compared to existing diagnostic tools. Since effective prevention requires more than accurate measurement, there is a growing need for more rigorous evaluation of the health benefits of using such devices.

The case of device-detected atrial fibrillation illustrates how the wellness technology industry’s *proactive* approach to making self-monitoring data key to prevention can drive a need for *reactive* research to determine whether the suggested strategy is beneficial or harmful. Such reactive research, like the LOOP study, can help improve guidelines and provide information about the spectrum of health conditions (such as arrhythmias) in different population groups. But while such research may help to identify “the right patients” in the future, this does not mean that what our informant called “unauthorized screening” is harmless in the present. Given uncertainties about health benefits, consumers may be turned into patients and be subjected to overdetection and overtreatment with medications that carry a risk of side effects. Meanwhile, the price for additional healthcare services and reactive research is paid from public resources, while manufacturers carry no burden of evidence to document health benefits prior or post marketing of their devices.

In the book *Drugs for Life: How Pharmaceutical Companies Define Our Health*, Dumit (2012) showed how pharmaceutical companies influence medical research, marketing, and diagnostic practices, leading to the normalization of lifelong medicine consumption. Similarly, the wellness technology industry may increasingly shape our understanding of health and illness through the message that staying healthy requires constant monitoring of invisible bodily signs via wearables. We may see these technologies as “devices for life”, which are marketed as life-long companions in the pursuit of a healthier life. Medicalization thus takes a specific shape in this context, as the monitoring itself comes with an expansion of the aspects of life that should be under medical surveillance. This has wide-ranging implications because the technologies entail a “technological intentionality”, encouraging users to not only to collect data but also perform health optimization in specific ways (Vegter et al. 2021). As the potential for health optimization is limitless, there is a perfect market for the wellness technology industry. But the risk of harmful effects raises important questions about the conditions under which it is ethically just for a screening program - whether authorized or “unauthorized” - to give people all the health information they want (Dive et al. 2023).

We have here focused on atrial fibrillation, but this is by no means a special case. The market for wellness technologies is growing rapidly. A variety of repurposed clinical tests and digital devices are becoming available directly to consumers, including tests for prostate health, thyroid function, testosterone levels, food sensitivity, HIV, and multiple forms of cancer, without evidence of health benefits (Gram et al. 2024; Muscat et al. 2025). Some companies also market blood tests for biological age as a way to increase longevity, although research to document the causal relationships between biomarkers and aging is still ongoing (Pinel et al. 2023). Meanwhile, FemTech companies specifically target women through co-opted feminist narratives, although the marketed hormone tests to inform reproductive choices are not evidence-based (Copp et al. 2024). Each of these applications comes with new opportunities to detect potential health problems but also with a range of new uncertainties that spill over as challenges for healthcare systems.

## Concluding remarks

The vision of “precision prevention” entails using advanced technologies to detect early signs of disease, in the hope that small-scale interventions can reduce downstream symptomatic disease states. This vision sounds promising, but early detection via new technologies often comes with downsides and challenges, often involving uneven distributions of benefits and harms. Wellness technologies, because of their regulatory status, are currently bypassing ethical and medical standards for what evidence is needed to ensure that health technologies are safe and beneficial. We have illustrated the challenge with the example of device-detected asymptomatic atrial fibrillation, which is currently highlighted by companies such as Apple and Huawei as empowering for both patients and healthcare providers. Yet, the LOOP study has shown that the lifesaving power of continuous heart monitoring is negligible, even for a high-risk population. There is thus a need for critical reflection on who pays and who benefits from precision prevention via wellness technologies.

The concerns raised by the GPs we interviewed, together with the findings of the LOOP study, highlight the complexities and risks of the implied alliance between the wellness technology industry and primary care. Precision prevention via new technologies can identify problems that require treatment, but it may come at the cost of overdiagnosing and overtreating many others. While consumer wearable devices promise early detection of conditions like atrial fibrillation, they often lack robust evidence of clinical benefit, leading to potential overdiagnosis, overtreatment, and increased healthcare costs. These devices, marketed as preventive tools, exploit regulatory gaps that allow manufacturers to avoid substantiating their claims, shifting the burden of evaluation and potential harms to users and healthcare systems.

In the loosely regulated sphere of consumer medicine, dubious claims about health benefits proliferate under the guise of patient empowerment. This raises difficult questions about how to balance unmet medical needs and respect for individual choice against what may be seen as paternalistic regulations that slow innovation. Yet, it is important not to conflate empowerment with unrestricted access to health data and diagnostic tools. At the moment, a burden of “reactive research”, overdiagnosis, and inefficient use of resources are likely to fall on consumers and public healthcare systems, while companies reap the profits. Thus, to counteract a situation where implementation precedes evidence, we call for protective measures requiring companies to provide evidence of health benefits *before* releasing everyday screening tools to the consumer market. Moreover, there is a need for regulatory oversight of trial design, data collection, and data sharing to properly document the achievement of their stated benefits.

The emergence of a new risk category, “device-detected subclinical atrial fibrillation,” underscores how wearables not only redefine disease and health through medicalization of everyday use of technologies and expanded disease definitions. The growing wellness technology industry also fast-tracks implementation of screening technologies prior to established evidence about benefits and harms. This temporal disruption of evidence comes at a substantial cost: patients risk unnecessary medicalization and treatment, while healthcare systems bear the financial and ethical burden of managing a population turned into unwitting research subjects. This dynamic, fueled by public expectations of the benefits of early detection, benefits device manufacturers while displacing responsibility for the consequences. While the pharmaceutical industry strategically has marketed the benefits of “drugs for life” (Dumit 2012), often through research efforts directed at their aims, “devices for life” are now marketed as life-long companions for health optimization while reshaping the landscape of medical research.

In concluding this paper, we encourage further cross-disciplinary reflection on the implications of the growing influence of big-tech companies in healthcare and beyond. By exploiting the current regulatory landscape, the marketing and uptake of wellness technologies demonstrate how ethical obligations in biomedical research can be sidestepped. The issue is not that big tech companies are conducting research without participants’ knowledge. Rather, what we scrutinize is that devices for early detection are marketed to consumers *before* robust evidence of safety and efficacy is produced, and that the company-sponsored studies are not designed to ensure production of sufficiently robust evidence of benefits and harms. Meanwhile, by driving the need for reactive studies, the wellness industry is also subtly reshaping the landscape of future medical research as well as how disease and health are detected and defined.

## Footnotes

1 Precision medicine is often referred to as “personalized medicine”, especially in Europe, although there is now an increasing preference for ”precision medicine” as a more scientifically neutral term (Chan and Erikainen 2018). We here use PM as an abbreviation for both, as the distinction is not crucial for our argument.

2 “Exploring Polygenic Risk Scores Using All of US”, https://allofus.nih.gov/article/exploring-polygenic-risk-scores-using-all-us, Published on June 6, 2024, accessed March 16, 2025.

3 While it is not our aim in this paper to discuss issues of data privacy and data safety, we find these issues equally important, particularly in insurance-based healthcare systems. On the topic of vested interests in health data economies, we have found Ebeling’s books on *Healthcare and Big Data* (2016) and *Afterlives of Data* (2022) particularly insightful.

4 The company Arivale was co-founded by Hood and Price and aimed to upscale the P100 project to 100.000 participants enrolled as consumers of the data-intensive approach documented in the P100 study. The company reached 5,000 paying customers but closed after 4 years due to difficulties acquiring enough customers to cover the costs. For further details, see “Why Arivale failed: Behind the scenes of first venture-backed medicine 3.0 company”, Healthy Debate by Max Marchione https://www.healthy-debate.com/p/arivale. Published March 11, 2024, Accessed Oct 6, 2025.

5 Overdiagnosis occurs when a diagnosis of disease or risk factor is irrelevant for health outcomes in terms of changes in morbidity or survival rates. This can for instance happen if abnormalities, such as cellular changes or small tumors, disappear spontaneously, or develop so slowly that the person remains asymptomatic until the person dies from other causes (Brodersen et al. 2018).

6 For further discussion on the gatekeeping function of Danish GPs, see (Spalletta and Green 2025).

7 “Heart health notifications on your Apple Watch”, https://support.apple.com/en-us/120276, accessed Jan 28, 2025.

8 The topic of digital health literacy is beyond the scope of this paper, but it is related to the difficulty of knowing whether, or not, to react to possible warning signs of wearable devices. For discussions on health literacy demands, gatekeeping, and low-value care, see (Muscat et al. 2025; Spalletta and Green 2025). For analyses of how GPs respond to patient-provided data and technologies in personalized medicine, see (Haase et al. 2023) and (Pot et al. 2024).

9 It should here be acknowledged that, in the Apple Heart Study, only 2161 participants received notifications of irregular pulse, which may dampen concerns about overdetection. Yet, more than half of the participants were between 22 and 39 years old (predominantly white males), while an older user population may be exposed to a higher risk of overdetection, even if they are of low risk of cardiac problems. Our criticism is thus not that the Apple Heart Study resulted in overdiagnosis (as we do not know this), but that this and similar studies are designed in a way that makes it impossible to properly evaluate the relationship between benefits and harms.

10 For examples, see Apple’s information on ECG, “Take an ECG with the ECG app on Apple Watch”: https://support.apple.com/en-us/120278, and Huawei’s user page on “ECG measurement on your HUAWEI watch“: https://consumer.huawei.com/en/support/content/en-us15888936/, both accessed October 6, 2025.
